# Association Between the Color Kanji Pick-Out Test App Performance and Cognitive Frailty as a Potential Early Screening Marker for Cognitive Decline

**DOI:** 10.3390/geriatrics11020041

**Published:** 2026-04-09

**Authors:** Akio Goda, Hideki Nakano, Yuki Kikuchi, Tsuyoshi Katsurasako, Kohei Mori, Atsuko Kubo, Kayoko Nonaka, Kohei Iwamoto, Nozomi Mitsumaru, Takaki Shimura, Shin Murata

**Affiliations:** 1 Hokuriku University Well-Being Research Team, Department of Physical Therapy, Faculty of Health and Medical Science, Hokuriku University, Kanazawa 920-1154, Japan; 2 Department of Physical Therapy, Faculty of Health Sciences, Kyoto Tachibana University, Kyoto 607-8175, Japan; 3 Tashiro Clinic, Keiyu-Kai Medical Corporation, Koka 528-0007, Japan; 4 Faculty of Allied Health Sciences, Kansai University of Welfare Sciences, Kashiwara 582-0026, Japan; 5 Faculty of Rehabilitation, Department of Rehabilitation, Nishikyushu University, Kanzaki 842-8585, Japan; 6 Department of Physical Therapy, Faculty of Rehabilitation, Reiwa Health Sciences University, Fukuoka 811-0213, Japan; 7 Department of Physical Therapy, Faculty of Rehabilitation Science, Kobe International University, Kobe 658-0032, Japan; 8 MIZ Co., Ltd., Saga 840-0054, Japan; 9 BME Research Laboratory, Sosei Ltd., Hamamatsu 432-8002, Japan

**Keywords:** cognitive frailty, reversible cognitive frailty, subjective cognitive decline, physical frailty (J-CHS criteria), Kihon Checklist (KCL), dual-task assessment, tablet-based cognitive screening, Stroop-like task, mild cognitive impairment (MCI)

## Abstract

**Background/Objective**: Cognitive frailty, the coexistence of physical frailty and cognitive impairment, is a potentially reversible and high-risk state for dementia. This study examined the association between Color Kanji Pick-out Test (CKPT) app performance and cognitive frailty independent of Mini-Mental State Examination (MMSE) scores in community-dwelling older women. **Methods**: In this cross-sectional study, the participants were 102 community-dwelling older women without dementia and with MMSE scores ≥ 27 (73.6 ± 6.0 years). Reversible cognitive frailty was defined as subjective cognitive decline (≥1 point in the cognitive domain of the Kihon Checklist) plus physical frailty or prefrailty, according to the Japanese Cardiovascular Health Study (J-CHS) criteria. Firth’s penalized logistic regression using three prespecified models, adjusted for age and education, was used to examine the independent associations between CKPT app performance and MMSE scores with reversible cognitive frailty. **Results**: Fourteen participants (13.7%) met the criteria for cognitive frailty. In separate models, higher CKPT app and MMSE scores were significantly associated with lower odds of cognitive frailty (CKPT: odds ratio [OR] 0.470, *p* = 0.019; MMSE: OR 0.548, *p* = 0.020). In a multivariable model including both measures, the CKPT app (OR 0.499, *p* = 0.031) and MMSE scores (OR 0.553, *p* = 0.031) remained independently associated with cognitive frailty, and this model had the lowest Akaike information criterion. **Conclusions**: The CKPT app performance was independently associated with cognitive frailty beyond global cognition. The CKPT app may detect subtle executive and attentional vulnerabilities not captured by the MMSE, supporting practical, objective, early screening and risk stratification of cognitive frailty.

## 1. Introduction

The acceleration of aging worldwide, including within Japan, has resulted in a marked increase in dementia prevalence, presenting a pressing social and medical challenge [1]. Dementia substantially impairs personal dignity and quality of life and imposes considerable economic and psychological burdens on caregivers and society at large [2]. Accordingly, there is a strong need to shift the paradigm from post-onset dementia care to prevention, by identifying individuals at risk in the prodromal stages, such as mild cognitive impairment (MCI) or even earlier [3].

In this context, cognitive frailty has attracted increasing attention as a high-risk state for dementia [4]. Cognitive frailty is generally defined as the coexistence of physical frailty and cognitive impairment in the absence of dementia and is associated with an increased risk of progression to dementia [5,6]. Ruan et al. [6] further proposed the concept of reversible cognitive frailty, characterized by the coexistence of physical frailty and subjective cognitive decline (SCD), as well as a narrower definition based solely on physical frailty and objective cognitive impairment (MCI). SCD is regarded as a pre-MCI condition [7] and has been identified as an early clinical marker of future dementia risk [8], even when standard neuropsychological test results remain within normal limits [9]. In community-based screening, the three-item cognitive domain of the Kihon Checklist (KCL-CF) is widely used as a practically viable tool to capture such subjective concerns, and its predictive validity for incident dementia has been established in large-scale cohort studies [10]. Furthermore, recent neurophysiological perspectives suggest that such subtle cognitive vulnerabilities may reflect changes in network-level neuroplasticity and cortical responsiveness, which precede overt structural brain changes. For instance, executive control processes, which are essential for navigating complex tasks, have been linked to specific event-related potential markers of frontal function [11], highlighting the importance of assessing integrated cognitive control mechanisms in early screening [12]. Accordingly, cognitive frailty is increasingly recognized as a multidimensional construct reflecting the complex interplay between neural vulnerability, systemic aging, and broader cognitive control mechanisms rather than isolated domain-specific deficits. Therefore, identifying individuals at risk at this stage and initiating timely interventions are essential elements of dementia prevention strategies.

Simple psychometrically sound assessment tools are required for widespread screening of reversible cognitive frailty among community-dwelling older adults. The cognitive domain of the KCL, which is widely used in Japan’s long-term care prevention programs, provides a brief self-report measure of SCD and has been shown to predict incident dementia [10,13,14,15]. However, self-report measures are susceptible to response biases [16]. In addition, interviewer-administered cognitive tests, such as the Mini-Mental State Examination (MMSE) [17], require trained assessors and are constrained by testing time and environment [18]. Therefore, additional objective measures are needed to detect subtle early changes in cognitive performance that can be routinely administered in community settings.

To address this gap, Goda et al. [19] developed the Color Kanji Pick-Out Test (CKPT) app, a tablet-based dual task that simultaneously assesses selective attention, processing speed, and short-term memory. The CKPT app requires participants to read a short story displayed on a tablet screen while identifying and tapping kanji characters that denote colors according to a Stroop-like rule, followed by multiple-choice recognition questions about the story content. A composite total score has been reported to detect subtle cognitive decline that may not be captured by the MMSE because of ceiling effects [19]. The app also incorporates automated voice guidance, scoring, and feedback, allowing assessments to be conducted without specialized staff and enabling large-scale community screenings.

However, the extent to which CKPT app performance reflects reversible cognitive frailty, a key target for dementia prevention in older adults, remains unclear. Previous studies have shown that SCD is associated with modifiable risk factors such as sleep disturbances and smoking [20]. However, the association between CKPT app performance and cognitive frailty, which integrates subjective cognitive complaints and physical frailty, has not been thoroughly examined.

Therefore, this study investigated whether CKPT app performance independently predicted the presence of reversible cognitive frailty, defined as the coexistence of physical frailty and SCD, among community-dwelling older women. This study focused exclusively on women to minimize sex-based variability in frailty phenotypes and physical performance [21]. Furthermore, we restricted the sample to individuals with MMSE scores ≥ 27 to specifically evaluate the CKPT app’s ability to detect cognitive vulnerabilities in a population typically screened as “cognitively normal” in routine clinical settings. Through these investigations, we evaluated the potential of the CKPT app as a simple and objective early screening tool that may contribute to extending healthy life expectancy in community settings.

## 2. Materials and Methods

### 2.1. Participants

This cross-sectional study used data collected from physical fitness assessment events for community-dwelling older adults held in Imari City (Saga Prefecture) and Koka City (Shiga Prefecture), Japan. The events were conducted annually between August and September, from 2021 to 2025. Recruitment notices were distributed through municipal newsletters, and the residents were invited to participate. A total of 531 community-dwelling older women attended the events. For individuals who participated in the assessment events in multiple years, only data from their first assessment were included in the analysis to ensure independence of observations.

Participants were included in the present analysis if they were aged ≥ 65 years and had an MMSE score ≥ 27 [22], indicating no evidence of cognitive impairment or suspected dementia. Participants were excluded if they had missing data for any variables used in the analysis or had physical or sensory impairments that could interfere with the assessments (e.g., neuromuscular, musculoskeletal, or cerebrovascular disease, major psychiatric disorders, or substantial visual or hearing impairment).

**Figure 1:**
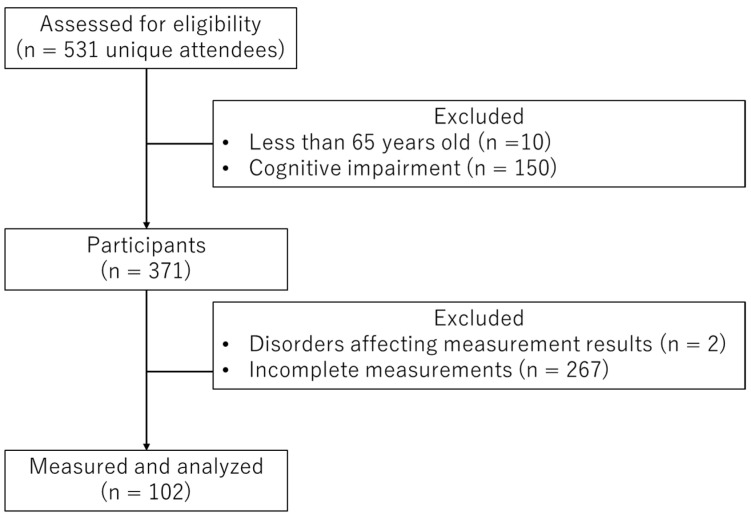
Flowchart of participant selection.

As shown in Figure 1, of the 531 unique attendees, we excluded those who were younger than 65 years old (*n* = 10) and those with cognitive impairment defined by an MMSE score < 27 (*n* = 150). Of the remaining 371 participants, we further excluded individuals with physical or sensory disorders affecting measurement results (*n* = 2) and those with incomplete data for any variables used in the analysis (*n* = 267). After applying these criteria, 102 women (mean age 73.55 ± 5.98 years) were included in the final analysis.

### 2.2. Ethical Considerations

The study was conducted in accordance with the Declaration of Helsinki and approved by the Ethics Committee of Kyoto Tachibana University (approval no. 18–26 and date of approval 18 July 2018). All participants received a full explanation of the study’s purpose and procedures, protection of personal information, and the right to withdraw at any time without disadvantage. Written informed consent was obtained from all the participants.

### 2.3. Measurements

In addition to the CKPT app, several other measures were also collected. Global cognitive function was assessed using the MMSE. Cognitive frailty (reversible cognitive frailty) was operationalized using the cognitive domain of the KCL and the Japanese version of the Cardiovascular Health Study (J-CHS) criteria for physical frailty. The demographic variables included age and years of education.

### 2.4. Color Kanji Pick-Out Test (CKPT) App

Early cognitive decline was assessed using the CKPT app [19]. The test was administered on a 12.9-inch iPad Pro (second generation, Apple Inc., Cupertino, CA, USA) with a stylus and headphones for audio instructions. Following automated voice guidance, participants silently read a short story presented on the screen while simultaneously performing a Stroop-like task: kanji characters denoting colors were presented in colored fonts, and participants were instructed to tap once when the character meaning and font color matched and twice when they did not match. The responses were recorded for 2 min. Immediately after the reading task, the participants answered three multiple-choice recognition questions about the story content. The primary CKPT app outcome was the total score, calculated as the number of correct color-judgment responses multiplied by the proportion of correct answers to the recognition questions; higher scores indicated better cognitive performance. Previously, the CKPT app’s acceptable test–retest reliability and its criterion validity was established, demonstrating significant correlations with conventional executive functioning measures such as the Wisconsin Card Sorting Test [19].

### 2.5. Mini-Mental State Examination (MMSE)

Global cognitive function was assessed using the MMSE [17]. The MMSE is a widely used brief screening test that evaluates multiple domains, including orientation, registration, attention and calculation, recall, language, and visuoconstruction skills. Total scores were calculated according to the standard procedures, with higher scores indicating better cognitive function. In line with a previous study [22], participants with MMSE scores ≥ 27 were considered to have normal cognition and were included in the present analysis.

### 2.6. Cognitive Frailty

Cognitive frailty was defined based on the concept of reversible cognitive frailty [23], as the coexistence of SCD and physical frailty status. SCD was assessed using the KCL-CF [13], which consists of three self-reported items: “Do your family or friends point out your memory loss (e.g., You keep asking the same thing)?,” “Do you make a call by looking up phone numbers?” and “Do you sometimes not know what the date is today?” Each item was scored as 0 or 1, and a total score of ≥1 (range 0–3) was considered indicative of SCD [20]. The KCL-CF has demonstrated acceptable validity [15] and reliability [14] as a screening tool for SCD in the general population.

Physical frailty was assessed using the J-CHS criteria [24], which comprise five components: unintentional weight loss, self-reported exhaustion, low physical activity, weak grip strength, and slow gait speed. In this study, each component was operationalized as follows: (1) weight loss was defined as an unintentional loss of ≥2 kg in the past 6 months; (2) exhaustion was defined as feeling exhausted without reason in the past 2 weeks; (3) low physical activity was defined as answering “no” to both engaging in light exercise/gymnastics and regular exercise/sports at least once a week; (4) weak grip strength was defined as <18 kg for women; and (5) slow gait speed was defined as <1.0 m/s. Classifications (robust, prefrail, or frail) were determined according to the J-CHS criteria; participants meeting three or more criteria were classified as frail, and those meeting one or two criteria were classified as prefrail. Grip strength was measured twice on each hand in a standing position using a digital handgrip dynamometer (T.K.K. 5401, Takei Scientific Instruments Co., Ltd., Niigata, Japan), and the highest value was used for the analyses. Gait speed was assessed as the usual walking speed over a 6 m walkway and calculated from the time required to walk the middle 4 m; the best of the two trials was used. Participants were classified as cognitively frail if they met the criteria for SCD (KCL-CF ≥ 1) and were classified as frail or pre-frail according to the J-CHS criteria.

### 2.7. Other Variables

Age (years) and total years of education were noted through interviews and included as covariates in the regression analyses.

### 2.8. Potential Sources of Bias and Mitigation Strategies

To ensure the robustness of the findings and minimize potential biases, several proactive measures were implemented. First, to address information bias, all assessments were conducted under standardized conditions. Specifically, the CKPT app was administered using identical tablet hardware (12.9-inch iPad Pro) and consistent audio-visual settings across all sites and years. All research staff underwent rigorous prior training to ensure uniformity in verbal instructions and physical performance measurements (e.g., grip strength and gait speed). Second, to mitigate site and year effects, the same core team of investigators supervised the data collection in both Imari and Koka cities, following a synchronized protocol from 2021 to 2025. Third, regarding selection bias, we acknowledge that participants were recruited from community fitness events, which may have attracted more health-conscious individuals (healthy volunteer bias). To minimize this impact, we recruited through broad municipal newsletters to reach a diverse range of residents, and applied strict inclusion criteria (MMSE ≥ 27) to focus on the target population for early screening.

### 2.9. Statistical Analysis

Descriptive statistics were calculated to summarize the participant characteristics. For descriptive completeness, both mean ± standard deviation (SD) and median (range) are presented for continuous variables. Regarding missing data, participants with incomplete measurements were excluded from the analysis (complete case analysis); the specific number of excluded cases is reported in Figure 1. Participants were then divided into those with cognitive frailty (*n* = 14) and those without (*n* = 88), and group differences were examined. For continuous variables (e.g., age, years of education, MMSE score, and CKPT app score), normality was assessed using the Shapiro–Wilk test. Variables with approximately normal distributions were compared using Student’s *t*-test, and non-normally distributed variables were compared using the Mann–Whitney *U* test. Categorical variables (e.g., SCD status and J-CHS categories) were compared using the chi-square test; when expected cell counts were <5, Fisher’s exact test was used (or the Fisher–Freeman–Halton exact test for variables with more than two categories).

To examine the association between CKPT app performance and cognitive frailty, Firth’s penalized logistic regression was performed with cognitive frailty status (yes/no) as the dependent variable. Although Poisson regression is often considered for outcomes with a prevalence > 10%, Firth’s approach was specifically selected to strictly control for small-sample bias [25,26,27] and separation issues given the limited number of cognitive frailty cases (*n* = 14). Age and years of education were included as covariates based on their established associations with cognitive function and frailty in the previous literature [28,29]. The explanatory variables were the CKPT app total score, MMSE score, age, and years of education. Continuous predictors were standardized (z-scores) prior to analysis so that the resulting odds ratios represent the change in odds associated with a 1-SD increase in each predictor, thereby allowing for the comparison of effect sizes across different cognitive measures. Three models were specified: Model 1 included the CKPT app score, age, and years of education; Model 2 included the MMSE score, age, and years of education; and Model 3 included the CKPT app score, MMSE score, age, and years of education. Additionally, preliminary analyses confirmed that neither the assessment site nor the year of data collection significantly confounded the primary associations; specifically, adding these variables to the models did not substantially alter the effect estimates for the CKPT app score, and they were thus excluded from the final models to maintain parsimony and avoid overfitting given the limited number of events. Odds ratios, 95% confidence intervals, and *p*-values were obtained from the profile likelihoods under Firth’s correction. The model fit was compared using the Akaike information criterion (AIC). Furthermore, to evaluate the incremental validity of the CKPT app over the MMSE, a penalized likelihood ratio test was conducted to compare the fits of Model 2 (MMSE and covariates) and Model 3 (CKPT app, MMSE, and covariates) using an identical sample. Multicollinearity was assessed using the variance inflation factor, with values < 2.0 considered acceptable. All analyses were conducted using IBM SPSS Statistics for Windows, version 31.0 (IBM Corp., Armonk, NY, USA) and R version 4.5.1 (R Foundation for Statistical Computing, Vienna, Austria). Specifically, the logistf package (version 1.26.1) was used for Firth’s penalized likelihood logistic regression, and the readxl package (version 1.4.5) was used for data importation. The significance level was set at *p* < 0.05.

## 3. Results

### 3.1. Participant Characteristics

Table 1 summarizes the characteristics of the 102 participants and compares those with (*n* = 14) and without cognitive frailty (*n* = 88). The mean age was 73.6 ± 6.0 years, and the mean years of education were 12.1 ± 1.6 years. Regarding the CKPT app, the total score distribution in the present sample approximated a normal distribution, with scores ranging from 0 to 17 (mean ± SD: 9.1 ± 3.9) points (95% confidence interval [CI]: 8.3–9.9). Fourteen participants (13.7%, 95% CI: 7.7–22.0%) met the criteria for cognitive frailty. MMSE scores and CKPT app total scores were significantly lower in the cognitive frailty group than in the no cognitive frailty group (*p* = 0.028 and *p* = 0.004, respectively). Age and years of education did not differ significantly between the groups (*p* = 0.065 and *p* = 0.390, respectively).

### 3.2. Association Between CKPT App Performance and Cognitive Frailty

Table 2 presents the results of the Firth’s penalized logistic regression analyses (odds ratios per 1-SD increase in each predictor). After adjusting for age and years of education, higher CKPT app total scores (Model 1: OR 0.470, 95% CI 0.236–0.886, *p* = 0.019) and higher MMSE scores were both associated with lower odds of cognitive frailty (Model 2: OR 0.548, 95% CI 0.322–0.909, *p* = 0.020). In the model including both cognitive measures (Model 3), the CKPT app total score (OR 0.499, 95% CI 0.250–0.938, *p* = 0.031) and MMSE score (OR 0.553, 95% CI 0.316–0.945, *p* = 0.031) remained independently associated with cognitive frailty. The magnitude of this standardized odds ratio indicates a substantial effect size for a 1-SD change in the CKPT score; however, the relatively wide 95% CI reflects the limited precision of this estimate owing to the small number of events. In contrast, age and years of education were not significant in any model. Model 3 had the lowest AIC (79.286), indicating a slightly better fit than Models 1 and 2. Importantly, the penalized likelihood ratio test demonstrated that adding the CKPT app total score to the model containing the MMSE score and covariates (Model 3) significantly improved model fit compared to the model containing only MMSE score and covariates (Model 2) (χ^2^ = 4.674, *p* = 0.031). This finding suggests the incremental value of the CKPT app in identifying cognitive frailty beyond global cognitive assessment.

## 4. Discussion

In this cross-sectional study of community-dwelling older women with normal MMSE scores, we examined the prevalence of cognitive frailty and the association between MMSE score and CKPT app performance, and cognitive frailty. Approximately 14% of the participants met the criteria for cognitive frailty. Higher MMSE and CKPT app scores were associated with lower odds of cognitive frailty, and these associations remained significant after mutual adjustment and after controlling for age and education. While the magnitude of the standardized odds ratio suggests a robust clinical association, the precision of the estimate was inevitably constrained by the limited sample size, highlighting the need to interpret the exact point estimate with appropriate caution. These findings suggest the potential value of combining a global cognitive screening test with a dual-task, tablet-based measure that places greater demands on executive and attentional processes for the early identification of cognitive frailty.

Group comparisons showed that women with cognitive frailty had significantly lower MMSE scores and CKPT app total scores than those without cognitive frailty, whereas age and years of education did not differ significantly between the groups. Notably, these differences were observed despite all participants scoring within the normal range on the MMSE (≥27). This pattern suggests that, in this sample, objective cognitive performance may be more closely associated with cognitive frailty status than with chronological age or educational attainment. Previous large-scale studies have reported independent associations between frailty severity and cognitive decline after adjusting for age and education, supporting the notion that frailty reflects clinically meaningful vulnerability beyond normal aging [30]. Our findings extend this literature by showing that CKPT app performance, which is intended to be sensitive to subtle cognitive changes, was lower among older women with cognitive frailty.

Firth’s penalized logistic regression further supported the independent association between CKPT app performance and MMSE scores with cognitive frailty status. In clinical and distributional terms, a 1-SD decrease in the CKPT app total score (approximately 3.9 points) corresponds roughly to a shift from the average performance level to the lower quartile of this study’s sample. This represents a meaningful decline in the integrated capacity for processing speed and accuracy. In models including each measure separately, higher scores on both tests were associated with lower odds of cognitive frailty. When both measures were entered simultaneously, each retained a significant association with cognitive frailty. This pattern suggests that the CKPT app captures aspects of cognition that are not fully captured by the MMSE [19,30]. Prior psychometric work has shown that CKPT app performance correlates with conventional measures of executive functioning, such as the Wisconsin Card Sorting Test (set shifting and error monitoring) [31], and with dual-task paradigms that tax attentional control [19]. These features are consistent with the design of the CKPT app as a Stroop-like dual task combined with memory demands. Importantly, rather than isolating specific cognitive domains such as processing speed or inhibitory control, the CKPT app’s complex demands are designed to capture broader cognitive control mechanisms and the overall capacity for attentional resource allocation. Since cognitive frailty is characterized by multidimensional neurocognitive vulnerabilities rather than single-domain deficits, the composite total score effectively reflects this integrated decline in executive-attentional networks [23]. Compared to traditional paper-based executive tests (e.g., Trail Making Test) [32] or standard physical dual tasks (e.g., walking while talking) [33], the CKPT app offers a scalable, standardized, and automated approach. Its specific added value in dementia-prevention strategies lies in its ability to efficiently detect these subtle executive-attentional vulnerabilities—core components of reversible cognitive frailty—before they manifest as overt functional decline, thereby providing a critical window for targeted interventions.

Cognitive frailty has been linked to vulnerabilities in executive functioning and attentional control rather than isolated deficits in a single cognitive domain [34]. Physically frail older adults often perform more poorly on tasks requiring sustained attention, divided attention, and flexible switching between mental sets [35,36,37,38]. In our study, the independent association of CKPT app performance with cognitive frailty, even among participants with relatively high MMSE scores, suggests that this dual-task measure may detect subtle, multidimensional changes in cognitive control that are not captured by the MMSE, which is known to have a ceiling effect in highly functioning older adults [18,39]. The slightly lower AIC for the model including both the CKPT app and MMSE indicates a marginally better model fit than models including either measure alone.

From a practical standpoint, these findings support the potential usefulness of incorporating the CKPT app into community-based screening programs for older adults. Because the app provides automated instructions and scoring and can be self-administered on a tablet, it may reduce the need for specialized staff and may be feasible for group screening or routine health checks [40]. Combining the CKPT app with brief global cognitive testing and physical frailty assessments may help identify older adults who may benefit from further assessment and timely interventions aimed at maintaining their cognitive and physical functions. Translated into a practical screening pathway, older adults who score within the normal range on standard global tests like the MMSE, but exhibit poor performance on the CKPT app, could be efficiently prioritized for more comprehensive geriatric assessments. In terms of public health implementation, the CKPT app could be seamlessly integrated into existing municipal screening workflows in Japan, for instance, by administering it alongside the KCL during annual community health check-ups. However, practical challenges must be considered. Older adults with low digital literacy or lack of familiarity with tablet devices may experience performance anxiety, potentially confounding their scores [41]. Indeed, during our data collection, while automated instructions were provided, a few participants required initial assistance from staff to familiarize themselves with the touch interface. Such varying needs for assistance could subtly influence performance and should be carefully considered in real-world screening contexts. Furthermore, the task heavily relies on visual and auditory processing; thus, appropriate accommodations or alternative assessments are necessary for individuals with significant visual or hearing limitations.

This study had several limitations. First, the cross-sectional design precludes conclusions about causal directionality or the directionality of the observed association between CKPT app performance and cognitive frailty. Second, the number of participants with cognitive frailty was relatively small. Although Firth’s penalized logistic regression reduces the small-sample bias by penalizing the likelihood function and provides more stable parameter estimates than standard logistic regression, the limited number of events still affects the precision of the estimates, as reflected in the relatively wide confidence intervals. Therefore, the observed associations, and particularly the apparent substantial effect size of the standardized odds ratios, must be interpreted cautiously to avoid potential overinterpretation, and our findings remain to be confirmed in larger cohorts. Third, our recruitment from community fitness events may have introduced selection bias, specifically a “healthy-volunteer bias,” as attendees of such events are likely to be more health-conscious or physically active than the general older population. Furthermore, a large proportion of initial attendees were excluded from the analysis because of incomplete measurements. Because complete demographic and clinical profiles were unavailable for these excluded individuals, we could not perform a direct statistical comparison between included and excluded participants, meaning that additional selection bias cannot be ruled out. Additionally, owing to the limited number of events (*n* = 14), we were unable to conduct sensitivity analyses using alternative SCD thresholds or physical frailty categorizations, which limits our ability to fully verify the robustness of this single definitional cut-off. Fourth, the inclusion was restricted to individuals with MMSE scores ≥ 27. While this allowed us to focus on subtle cognitive decline, such range restriction might have led to an underestimation of the strength of the association between global cognition and cognitive frailty. Fifth, the sample consisted exclusively of community-dwelling older women; therefore, it is unclear whether similar associations would be observed in men, in more frail or institutionalized populations, or in more diverse populations. Additionally, although we standardized the assessment protocols across sites and years, potential site-specific or year-specific effects during the 2021–2025 data collection period cannot be entirely ruled out. Finally, cognitive assessment was limited to the MMSE and CKPT app; we did not administer a comprehensive neuropsychological battery to examine domain-specific associations. Furthermore, we did not evaluate other potential confounding factors, such as depression [42], sleep quality [43], and medication use [44], which are known to influence both cognitive performance and frailty. The absence of these variables means that the observed independent associations must be interpreted with caution. Moreover, because the CKPT app requires Kanji reading comprehension and basic tablet familiarity, its applicability to populations with varying literacy levels, digital skills, or different cultural settings remains to be investigated [41]. Future longitudinal studies with larger, more diverse samples and broader cognitive assessments are needed to confirm the predictive validity of CKPT app performance for incident cognitive frailty and dementia and to clarify the underlying cognitive mechanisms of these relationships.

## 5. Conclusions

Among community-dwelling older women with normal MMSE scores, lower performance on the tablet-based CKPT app was independently associated with cognitive frailty (reversible cognitive frailty) after adjusting for age, education, and MMSE score. The independent association of the CKPT app score in multivariable models suggests that it captures vulnerabilities in processing speed, attentional control, and executive function that are not fully reflected in the MMSE. In community screening settings, combining the CKPT app with conventional assessments may facilitate the early identification of older adults with cognitive frailty and support timely referral for physical and cognitive interventions. Longitudinal studies are needed to determine whether CKPT app performance predicts the future onset or progression of cognitive frailty and to clarify its potential role in dementia prevention strategies.

## Figures and Tables

**Table 1:** Characteristics of participants and comparisons between those with and without cognitive frailty.

Variable	Total (*n* = 102)	Cognitive Frailty (*n* = 14)	No Cognitive Frailty (*n* = 88)	*p*-Value
Age (years) ^†^	73.55 ± 5.98; 73 (60–89)	76.29 ± 5.76; 78 (66–83)	73.11 ± 5.93; 73 (60–89)	0.065
Years of education ^‡^	12.07 ± 1.64; 12 (8–18)	11.71 ± 1.44; 12 (9–15)	12.13 ± 1.67; 12 (8–18)	0.390
MMSE score ^‡^	29.42 ± 0.79; 30 (27–30)	28.86 ± 1.17; 29 (27–30)	29.51 ± 0.68; 30 (28–30)	0.028
CKPT app total score ^†^	9.10 ± 3.85; 8.82 (0–17)	6.36 ± 3.12; 6.25 (1.6–12)	9.54 ± 3.79; 10 (0–17)	0.004
Subjective cognitive decline, *n* (%)				<0.001
Present	24 (23.5%)	14 (100%)	10 (11.4%)	
Absent	78 (76.5%)	0 (0%)	78 (88.6%)	
No. of KCL cognitive items endorsed ^‡^	0.31 ± 0.61; 0 (0–2)	1.50 ± 0.52; 1.5 (1–2)	0.13 ± 0.37; 0 (0–2)	<0.001
“Family or friends say you ask the same things repeatedly”, *n* (%) (Present/Absent)				<0.001
Present	11 (10.8%)	8 (57.1%)	3 (3.4%)	
Absent	91 (89.2%)	6 (42.9%)	85 (96.6%)	
“You are unable to make a call by looking up phone numbers.”, *n* (%) (Present/Absent)				<0.001
Present	6 (5.9%)	5 (35.7%)	1 (1.1%)	
Absent	96 (94.1%)	9 (64.3%)	87 (98.9%)	
“You sometimes do not know today’s date”, *n* (%) (Present/Absent)				<0.001
Present	15 (14.7%)	8 (57.1%)	7 (8.0%)	
Absent	87 (85.3%)	6 (42.9%)	81 (92.0%)	
J-CHS classification (frail/prefrail/robust), *n* (%) *				<0.001
Frail	5 (4.9%)	3 (21.4%)	2 (2.3%)	
Prefrail	37 (36.3%)	11 (78.6%)	26 (29.5%)	
Robust	60 (58.8%)	0 (0.0%)	60 (68.2%)	
No. of J-CHS criteria met ^‡^	0.60 ± 0.87; 0 (0–4)	1.64 ± 1.01; 1 (1–4)	0.43 ± 0.72; 0 (0–3)	<0.001
Weight loss, *n* (%) (Present/Absent)				0.227
Present	16 (15.7%)	4 (28.6%)	12 (13.6%)	
Absent	86 (84.3%)	10 (71.4%)	76 (86.4%)	
Weak grip strength, *n* (%) (Present/Absent)				0.001
Present	11 (10.8%)	6 (42.9%)	5 (5.7%)	
Absent	91 (89.2%)	8 (57.1%)	83 (94.3%)	
Exhaustion, *n* (%) (Present/Absent)				0.001
Present	21 (20.6%)	8 (57.1%)	13 (14.8%)	
Absent	81 (79.4%)	6 (42.9%)	75 (85.2%)	
Slow gait speed, *n* (%) (Present/Absent)				0.245
Present	7 (6.9%)	2 (14.3%)	5 (5.7%)	
Absent	95 (93.1%)	12 (85.7%)	83 (94.3%)	
Low physical activity, *n* (%) (Present/Absent)				0.033
Present	6 (5.9%)	3 (21.4%)	3 (3.4%)	
Absent	96 (94.1%)	11 (78.6%)	85 (96.6%)	

Chi-square test. ^†^ Parametric test (Student’s *t*-test); ^‡^ Non-parametric test (Mann–Whitney *U* test); * Fisher–Freeman–Halton exact test. MMSE, Mini-Mental State Examination; CKPT, Color Kanji Pick-out Test; KCL, Kihon Checklist; J-CHS, Japanese version of the Cardiovascular Health Study criteria. The phone-call item was reverse-coded for consistency with the Present/Absent format.

**Table 2:** Firth’s penalized logistic regression models for cognitive frailty (yes/no).

Model	Variable	β_Std (Standardized Coefficient)	Odds Ratio (per 1 SD)	95% CI (Lower–Upper)	*p*-Value	AIC
Model 1	CKPT app total score	−0.755	0.470	0.236–0.886	0.019	79.798
	Age (years)	0.261	1.298	0.694–2.513	0.417	
	Years of education	0.100	1.105	0.572–2.056	0.759	
Model 2	MMSE score	−0.601	0.548	0.322–0.909	0.020	79.670
	Age (years)	0.282	1.326	0.739–2.475	0.347	
	Years of education	−0.189	0.828	0.403–1.659	0.601	
Model 3	CKPT app total score	−0.695	0.499	0.250–0.938	0.031	79.286
	MMSE score	−0.592	0.553	0.316–0.945	0.031	
	Age (years)	0.018	1.018	0.518–2.013	0.959	
	Years of education	−0.047	0.954	0.450–1.882	0.898	

Firth’s penalized logistic regression. Estimates, *p*-values, and 95% confidence intervals were obtained from the profile likelihoods under Firth’s correction. The AIC was calculated from the non-penalized (ordinary) log-likelihood ℓ as AIC = −2ℓ + 2k, where k is the number of estimated parameters, including the intercept. CKPT, Color Kanji Pick-out Test; MMSE, Mini-Mental State Examination; SD, standard deviation; CI, confidence interval; AIC, Akaike information criterion.

## Data Availability

The datasets presented in this article are not readily available, because the data are part of an ongoing study. Requests to access the datasets should be directed to the correspondence author.

## Abbreviations

The following abbreviations are used in this manuscript:

AIC	Akaike information criterion
CKPT	Color Kanji Pick-out Test
MCI	Mild cognitive impairment
MMSE	Mini-Mental State Examination
SCD	Subjective cognitive decline

## References

[1] Ishihara M., Matsunaga S., Islam R., Shibata O., Chung U.I. (2024). A policy overview of Japan’s progress on dementia care in a super-aged society and future challenges. Glob. Health Med..

[2] Tahami Monfared A.A., Khachatryan A., Hummel N., Kopiec A., Martinez M., Zhang R., Zhang Q. (2024). Assessing Quality of Life, Economic Burden, and Independence Across the Alzheimer’s Disease Continuum Using Patient-Caregiver Dyad Surveys. J. Alzheimer’s Dis..

[3] Rafii M.S., Aisen P.S. (2023). Detection and treatment of Alzheimer’s disease in its preclinical stage. Nat. Aging.

[4] Yamasaki T., Tanaka M., Kumagai S. (2025). Editorial: Recent advances in research on cognitive frailty and related conditions. Front. Aging Neurosci..

[5] Kelaiditi E., Cesari M., Canevelli M., van Kan G.A., Ousset P.J., Gillette-Guyonnet S., Ritz P., Duveau F., Soto M.E., Provencher V. (2013). Cognitive frailty: Rational and definition from an (I.A.N.A./I.A.G.G.) international consensus group. J. Nutr. Health Aging.

[6] Ruan Q., Yu Z., Chen M., Bao Z., Li J., He W. (2015). Cognitive frailty, a novel target for the prevention of elderly dependency. Ageing Res. Rev..

[7] Jessen F., Amariglio R.E., van Boxtel M., Breteler M., Ceccaldi M., Chételat G., Dubois B., Dufouil C., Ellis K.A., van der Flier W.M. (2014). A conceptual framework for research on subjective cognitive decline in preclinical Alzheimer’s disease. Alzheimer’s Dement..

[8] Jessen F., Amariglio R.E., Buckley R.F., van der Flier W.M., Han Y., Molinuevo J.L., Rabin L., Rentz D.M., Rodriguez-Gomez O., Saykin A.J. (2020). The characterisation of subjective cognitive decline. Lancet Neurol..

[9] Whitfield T., Chouliaras L., Morrell R., Rubio D., Radford D., Marchant N.L., Walker Z. (2024). The criteria used to rule out mild cognitive impairment impact dementia incidence rates in subjective cognitive decline. Alzheimer’s Res. Ther..

[10] Tomata Y., Sugiyama K., Kaiho Y., Sugawara Y., Hozawa A., Tsuji I. (2017). Predictive ability of a simple subjective memory complaints scale for incident dementia: Evaluation of Japan’s national checklist, the “Kihon Checklist”. Geriatr. Gerontol. Int..

[11] Folstein J.R., Van Petten C. (2008). Influence of cognitive control and mismatch on the N2 component of the ERP: A review. Psychophysiology.

[12] Di Fazio C., Scaliti E., Stanziano M., Nigri A., Demichelis G., Tamietto M., Palermo S. (2026). Exploring cortical excitability modulation to promote cognitive resilience in aging: An rTMS study protocol. Front. Hum. Neurosci..

[13] Okura M., Ogita M., Arai H. (2019). Self-Reported Cognitive Frailty Predicts Adverse Health Outcomes for Community-Dwelling Older Adults Based on an Analysis of Sex and Age. J. Nutr. Health Aging.

[14] Sampaio P., Sampaio R.A.C., Yamada M., Arai H. (2016). Systematic review of the Kihon Checklist: Is it a reliable assessment of frailty?: Kihon Checklist: Systematic review. Geriatr. Gerontol. Int..

[15] Satake S., Senda K., Hong Y.J., Miura H., Endo H., Sakurai T., Kondo I., Toba K. (2016). Validity of the Kihon Checklist for assessing frailty status. Geriatr. Gerontol. Int..

[16] Goldberg S.M., Lopez O.L., Cohen A.D., Klunk W.E., Aizenstein H.A., Mizuno A., Snitz B.E. (2021). The roles of study setting, response bias, and personality in subjective memory complaints of cognitively normal older adults. Int. Psychogeriatr..

[17] Folstein M.F., Folstein S.E., McHugh P.R. (1975). ‘Mini-mental state’. A practical method for grading the cognitive state of pa-tients for the clinician. J. Psychiatr. Res..

[18] Mitchell A.J. (2009). A meta-analysis of the accuracy of the mini-mental state examination in the detection of dementia and mild cognitive impairment. J. Psychiatr. Res..

[19] Goda A., Shimura T., Murata S., Abiko T., Miyachi R., Ohsugi H., Okuyama E. (2023). Development of an Application for Assessment of Very Early Stage Cognitive Decline. J. New Med. Innov. Res..

[20] Goda A., Nakano H., Kikuchi Y., Mori K., Mitsumaru N., Murata S. (2024). Association between Subjective Cognitive Complaints and Sleep Disturbance among Community-Dwelling Elderly Individuals in Japan. Healthcare.

[21] Gordon E.H., Peel N.M., Samanta M., Theou O., Howlett S.E., Hubbard R.E. (2017). Sex differences in frailty: A systematic review and meta-analysis. Exp. Gerontol..

[22] Hatanaka S., Sasai H., Shida T., Osuka Y., Kojima N., Ohta T., Abe T., Yamashita M., Obuchi S.P., Ishizaki T. (2024). Association between dynapenia and cognitive decline in community-dwelling older Japanese adults: The IRIDE Cohort Study. Geriatr. Gerontol. Int..

[23] Sugimoto T., Arai H., Sakurai T. (2022). An update on cognitive frailty: Its definition, impact, associated factors and underlying mechanisms, and interventions. Geriatr. Gerontol. Int..

[24] Satake S., Arai H. (2020). The revised Japanese version of the Cardiovascular Health Study criteria (revised J-CHS criteria). Geriatr. Gerontol. Int..

[25] Barros A.J., Hirakata V.N. (2003). Alternatives for logistic regression in cross-sectional studies: An empirical comparison of models that directly estimate the prevalence ratio. BMC Med. Res. Methodol..

[26] Firth D. (1993). Bias Reduction of Maximum Likelihood Estimates. Biometrika.

[27] Heinze G., Schemper M. (2002). A solution to the problem of separation in logistic regression. Stat. Med..

[28] Harada C.N., Natelson Love M.C., Triebel K.L. (2013). Normal cognitive aging. Clin. Geriatr. Med..

[29] Hoogendijk E.O., van Hout H.P., Heymans M.W., van der Horst H.E., Frijters D.H., Broese van Groenou M.I., Deeg D.J., Huisman M. (2014). Explaining the association between educational level and frailty in older adults: Results from a 13-year longitudinal study in The Netherlands. Ann. Epidemiol..

[30] García-Chanes R.E., Avila-Funes J.A., Borda M.G., Pérez-Zepeda M.U., Gutiérrez-Robledo L.M. (2023). Higher frailty levels are associated with lower cognitive test scores in a multi-country study: Evidence from the study on global ageing and adult health. Front. Med..

[31] Barceló F., Sanz M., Molina V., Rubia F.J. (1997). The Wisconsin Card Sorting Test and the assessment of frontal function: A validation study with event-related potentials. Neuropsychologia.

[32] Bowie C.R., Harvey P.D. (2006). Administration and interpretation of the Trail Making Test. Nat. Protoc..

[33] Ceïde M.E., Ayers E.I., Lipton R., Verghese J. (2018). Walking While Talking and Risk of Incident Dementia. Am. J. Geriatr. Psychiatry.

[34] Bartoli M., Palermo S., Cipriani G.E., Amanzio M. (2020). A Possible Association Between Executive Dysfunction and Frailty in Patients with Neurocognitive Disorders. Front. Psychol..

[35] Gross A.L., Xue Q.L., Bandeen-Roche K., Fried L.P., Varadhan R., McAdams-DeMarco M.A., Walston J., Carlson M.C. (2016). Declines and Impairment in Executive Function Predict Onset of Physical Frailty. J. Gerontol. A Biol. Sci. Med. Sci..

[36] O’Halloran A.M., Fan C.W., Kenny R.A., Pénard N., Galli A., Robertson I.H. (2011). Variability in sustained attention and risk of frailty. J. Am. Geriatr. Soc..

[37] O’Halloran A.M., Finucane C., Savva G.M., Robertson I.H., Kenny R.A. (2014). Sustained attention and frailty in the older adult population. J. Gerontol. B Psychol. Sci. Soc. Sci..

[38] Rosado-Artalejo C., Carnicero J.A., Losa-Reyna J., Castillo C., Cobos-Antoranz B., Alfaro-Acha A., Rodríguez-Mañas L., García-García F.J. (2017). Global Performance of Executive Function Is Predictor of Risk of Frailty and Disability in Older Adults. J. Nutr. Health Aging.

[39] Franco-Marina F., García-González J.J., Wagner-Echeagaray F., Gallo J., Ugalde O., Sánchez-García S., Espinel-Bermúdez C., Juárez-Cedillo T., Rodríguez M.Á.V., García-Peña C. (2010). The Mini-mental State Examination revisited: Ceiling and floor effects after score adjustment for educational level in an aging Mexican population. Int. Psychogeriatr..

[40] Tsoy E., Zygouris S., Possin K.L. (2021). Current State of Self-Administered Brief Computerized Cognitive Assessments for Detection of Cognitive Disorders in Older Adults: A Systematic Review. J. Prev. Alzheimer’s Dis..

[41] Pellicer-Espinosa I., Díaz-Orueta U. (2022). Cognitive Screening Instruments for Older Adults with Low Educational and Literacy Levels: A Systematic Review. J. Appl. Gerontol..

[42] Zou C., Yu Q., Wang C., Ding M., Chen L. (2023). Association of depression with cognitive frailty: A systematic review and meta-analysis. J. Affect. Disord..

[43] Kaur S., Banerjee N., Miranda M., Slugh M., Sun-Suslow N., McInerney K.F., Sun X., Ramos A.R., Rundek T., Sacco R.L. (2019). Sleep quality mediates the relationship between frailty and cognitive dysfunction in non-demented middle aged to older adults. Int. Psychogeriatr..

[44] Moon J.H., Huh J.S., Won C.W., Kim H.J. (2019). Is Polypharmacy Associated with Cognitive Frailty in the Elderly? Results from the Korean Frailty and Aging Cohort Study. J. Nutr. Health Aging.

